# Exploring integrated advice and avian influenza knowledge gaps by using a Highly Pathogenic Avian Influenza outbreak simulation

**DOI:** 10.1371/journal.pone.0354920

**Published:** 2026-08-26

**Authors:** Femke W. Overbosch, Charlotte N. M. Waltz, Tomris Cesuroglu, Bart Blokland, Jeannette de Boer, Pearl A. Dykstra, Danielle R. M. Timmermans, Bas ter Weel, Marijn de Bruin, Marion P. G. Koopmans, Anja J. M. Schreijer

**Affiliations:** 1 Erasmus Medical Center, Pandemic and Disaster Preparedness Center, Rotterdam, the Netherlands; 2 Department of Public Administration and Sociology, Erasmus School of Social and Behavioural Sciences, Erasmus University Rotterdam, Rotterdam, the Netherlands; 3 Department of Public and Occupational Health, Amsterdam Public Health Research Institute, Amsterdam UMC, Vrije Universiteit Amsterdam, Amsterdam, the Netherlands; 4 SEO Amsterdam Economics, Amsterdam, the Netherlands; 5 Faculty of Economics and Business, section Microeconomics, University of Amsterdam, Amsterdam, the Netherlands; 6 Department Behaviour and Health, National Institute for Public Health and the Environment (RIVM), Bilthoven, the Netherlands; 7 Department IQ Health, Institute of Health Sciences, Radboud University Medical Center, Nijmegen, the Netherlands; 8 Department of Viroscience, Erasmus MC, Rotterdam, the Netherlands; Central Laboratory for Evaluation of Veterinary Biologics, Agricultural Research Center, EGYPT

## Abstract

**Background:**

Initial evaluations of the COVID-19 pandemic suggested that broad multidisciplinary expertise is needed for an all of society pandemic response. However, it is unclear whether balanced, multi-perspective scientific advising is secured in separate disciplinary advisory committees.

**Aim:**

Our aim was to explore the potential of integrated advising among biomedical, societal and economic experts into pandemic response, using a simulation of a highly pathogenic avian influenza (HPAI) outbreak. Additionally, we aimed to identify cross-cutting gaps in knowledge and expertise relevant for HPAI pandemic preparedness.

**Methods:**

We conducted two table-top simulation exercises (April/May 2024), based on a fictitious HPAI outbreak originating in the Netherlands and associated with severe disease among children. In total, 22 and 19 experts participated, representing biomedical, societal and economic sciences. Participants first drafted an outbreak management advice in separate biomedical and societal teams, followed by integrated deliberations and advice in mixed groups. Written notes of the deliberations and pre-simulation interviews was used for an inductive, reflexive thematic analysis.

**Results:**

Separate advisory processes differed in perceived urgency, situation assessment, and the drafted best- and worst-case scenarios. Mixed groups deliberations exposed disciplinary blind spots and fostered mutual understanding. In particular, school closure was discussed extensively due to conflicting perspectives. Cross-cutting knowledge and know-how gaps were identified in the alignment of (infra)structures and guidelines.

**Conclusions:**

Our simulation exercises suggest that an integrated advisory approach may have an added value in overcoming conflicting advice during pandemics. Further research is needed to explore whether this observed added value extends to an integrated advisory body.

## Introduction

With growing pressure on ecosystems, an increasing risk of spillovers of pathogens from animals to humans, and multiple global changes that may influence the dynamics of infectious diseases, there is a constant risk of a new infectious disease outbreak or pandemic [1]. Early recognition coupled with prompt response to an emerging pathogen is imperative as this can prevent or mitigate a (larger) public health crisis. Public health measures recommended during the COVID-19 pandemic were associated with potential detrimental societal and economic impact, and the need to mitigate these effects was increasingly recognized. [2–9]. While the need for multidisciplinary advice as part of pandemic preparedness had been recognized prior to the COVID-19 pandemic, few countries had broad expertise from scientific domains beyond biomedical sciences in their advisory bodies [10,11].

In The Netherlands, since 1995, the Dutch Outbreak Management Team (OMT) -consisting of a core of experts in infectious disease preparedness, treatment and control- has advised the Minister of Health in the event of an outbreak of concern, including before and during the COVID-19 pandemic [12]. An OMT zoonoses including an extended zoonoses structure is in place in case of zoonotic threats [13]. While many additional experts from other disciplines have been recruited for the OMT depending on the outbreak, such as social science experts, the focus of the OMT has been primarily biomedical. Logically, the OMT was not sufficiently equipped to provide the necessary recommendations to limit the societal and economic impact of the long-lasting COVID-19 outbreak.

**Fig 1:**
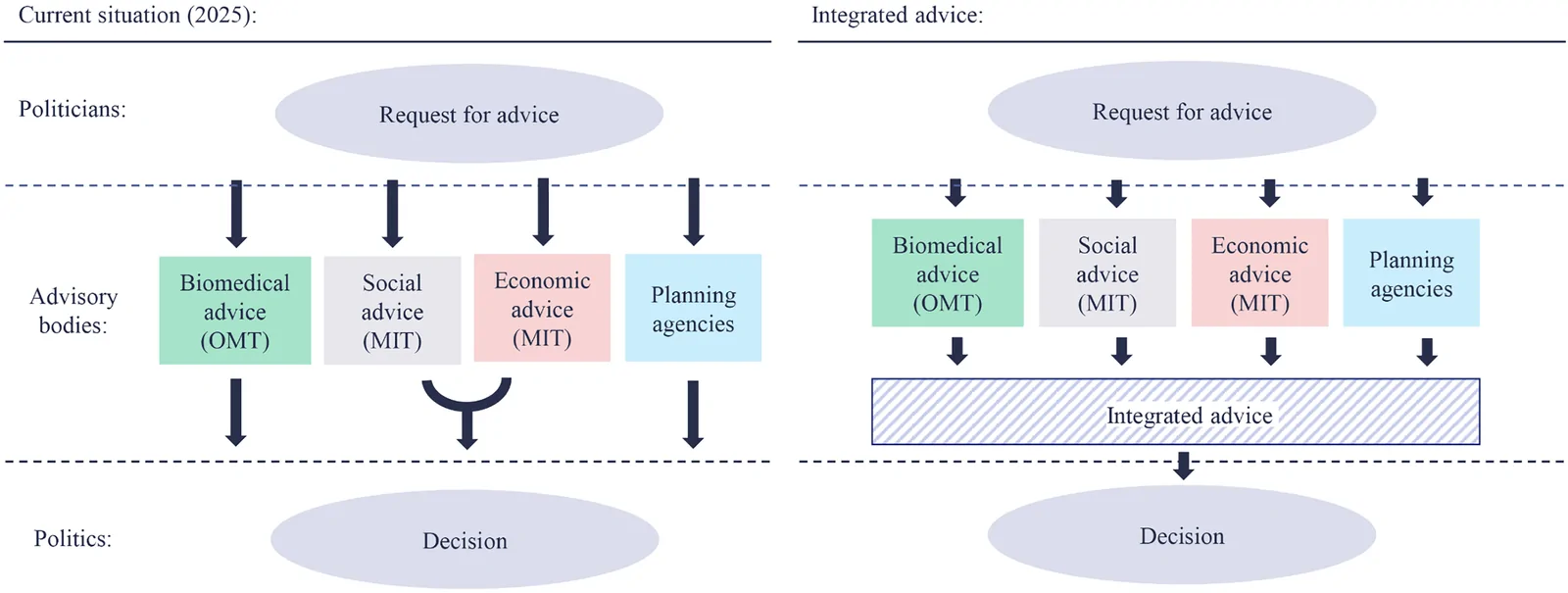
Left: Current prevailed form of advising bodies during an infectious disease outbreak of (inter)national concern in the Netherlands; right: proposition of an alternative advising approach which needs to be further investigated [15].

Therefore, next to the valuable biomedical advice, social, behavioral and economic advice was provided by multiple existing official government advisory bodies such as the National Institute for Social Research (SCP), Netherlands Bureau for Economic Policy Analysis (CPB), and the behavioral unit of the National Institute for Public Health and the Environment (RIVM) during the COVID-19 pandemic. When the pandemic evolved, an early attempt was made to harmonize societal, behavioral and economic advice in a newly developed Societal Impact Team (in Dutch ‘Maatschappelijk Impact Team’ or MIT). This team consisting of societal and economic experts, provided advice to the Minister of Social Affairs and Employment from 1 September 2022 on wards [14]. The OMT and the MIT separately advised the ministers during the later phase of the COVID-19 pandemic. The two ministries were tasked with a balanced decision and implementation of the separate advised public health measures (see Fig 1, left). However, without exchange between advisory bodies, there is a risk that recommended measures will be presented in a one-sided manner or may even be conflicting. This could lead to unbalanced advice, resulting in a sub optimal outbreak management policy.

In the post-COVID-19 period, multiple evaluation reports and papers have been published with lessons learned from the COVID-19 pandemic about the pathogen, interventions, measures, accessibility, infrastructures, data collection et cetera [16–22]. These lessons may provide valuable insights to optimize outbreak management strategies and prepare for new pandemic threats. To also learn from the used advisory structures, as a pilot study, we recently conducted a multidisciplinary table-top exercise to evaluate the expert advice provided during two specific phases of the COVID-19 pandemic [6]. This study concluded that, compared to the current prevailed situation (Fig 1 left panel), an integrated advice can provide a multi-perspective substantiation of an advice request and might potentially provide better quality of support to policy makers (see Fig 1 right panel).

Here, our aim was to further explore the organization of such integrated advice in the context of a current threat with pandemic potential, using an outbreak simulation exercise as a basis. H5N1 Highly pathogenic avian influenza (HPAI) has been detected in several mammalian species, including goats and dairy cattle in the USA in 2024, and recently in kittens in the Netherlands [23,24]. Suspected and confirmed cases of HPAI in wild and domestic birds are monitored very closely in the Netherlands [25]. In the event of a confirmed case among domestic poultry in the Netherlands, with its densely populated intensive poultry farming industry, strict measures are taken [25]. Despite the limited number of human cases worldwide, the WHO has placed HPAI on its list of priority pathogens and considers the risk of a public health emergency of international concern caused by HPAI to be high [26,27].

We conducted two table-top simulation exercises of a fictitious but realistic HPAI outbreak originating in the Netherlands. The fictitious outbreak occurred in two pandemic phases, corresponding to WHO pandemic influenza phases 4 and 6, respectively-(supplement S1 Fig), requiring different levels of response intensity. To develop an interdisciplinary HPAI knowledge agenda, we sought to understand key knowledge and process concepts, data and analyses that would need to be considered for policy advice. We addressed the following exploratory research questions:

1. What key knowledge and know-how (the knowledge of how to implement expertise or methods) are provided through the separate biomedical and socio-economic expert groups?
2. Are there topics where biomedical and socio-economic expert advice may lead to conflicting recommendations?
3. Are there characteristics of separate biomedical and socio-economic expert advice that may lead to discord in the advice process?
4. Can we identify key themes that contribute to the alignment of conflicting recommendations or process?
5. What are domain-overarching knowledge and know-how gaps that contribute to integrated HPAI pandemic preparedness?

## Methods

We conducted two tabletop simulation exercises (i.e., simulated emergency situations designed to facilitate discussion among participants) regarding an HPAI sub-type H5N1 outbreak originating in wild birds and with a spill-over to pigs and humans in the Netherlands. Dutch experts from various disciplines were recruited through purposive sampling from the existing network of the research group, and complemented by targeted invitations. Criteria to invite an expert to participate were: 1) the invited experts represented disciplines which can be requested into a scientific advisory committee in a case of an avian influenza outbreak such as virology, veterinary sciences, epidemiology, public health, economics, sociology, communication, and behavioral sciences, 2) the expert has demonstrable experience in his/her field of expertise (e.g., principal investigator or professor) and/or 3) has experience as a member of an Outbreak Management Team or Societal team advising the Dutch government during COVID-19 or previous large outbreaks. To prevent over-representation during the exercises, only one or two experts from a similar discipline were invited. To prevent disbalances or under-representation of disciplines, new invites were send to other experts within a similar discipline upon a decline or non-response of an eligible participant. Optimal group size for deliberation was considered at eight to ten participants per group.

### Design tabletop simulation exercises

The simulation exercises were held in the Netherlands on 17 April and 24 May 2024, and each consisted of a full-day program. The exercises consisted of introductory presentations, a morning session in which participants were grouped according to their disciplines (mimicking the current prevailing governance framework on scientific advice during outbreaks in two separate bodies), and an afternoon session in which participants were mixed in two or three groups respectively. A modified WHO-Integrate COVID-19 (WICID) Evidence-to-Decision framework was used for integrated advice discussions [28]. Briefly, the framework guides systemic reflection on interventions within their specific contexts using eleven different criteria such as health benefits and harms: equity, equality and feasibility and implementation. No specific criteria were set for the integrated advice, providing each mixed group the liberty to explore a preferred joint approach – whether consensus, merging recommendations or substantiating (separate) recommendations, or a combination thereof – for the additional integrated advice. Each group session was followed by a plenary reflection and each simulation exercise was completed with a reflection and evaluation session.

The (background) presentations provided the participating experts with biomedical, social, behavioral and economic information about the simulation scenario.

In the morning exercise, the experts from the virology, veterinary sciences, epidemiology and public health were united in the biomedical team. The experts of the economics, sociology, communication and behavioral sciences were united in a societal team. The biomedical and societal team were invited to respond separately to a request for advice from the Ministry of Health. This request had been formulated ahead of the simulation exercise by representatives of the Ministry of Health based on the simulation scenario. The request included questions and sub-questions regarding the policy goal ‘guard healthcare access for the entire population’ and ‘maintain social and economic continuity and vitality.’

**Fig 2:**
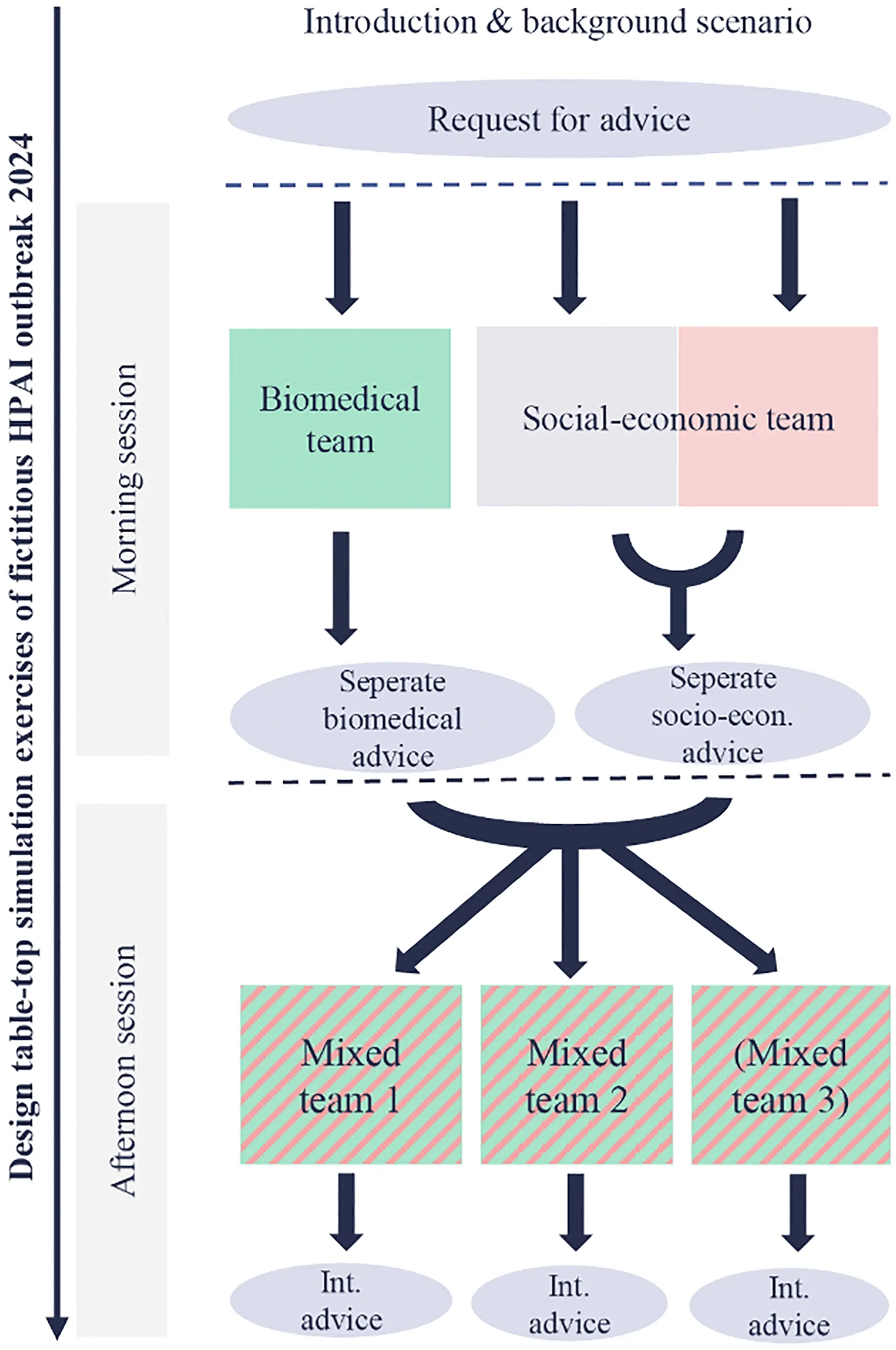
Design of the table-top simulation exercises of the fictitious HPAI outbreak.

In the afternoon session, participants regrouped into mixed teams of biomedical and socio-economic scientists and the draft advice from the morning session were provided to all participants in printed form. Participants were then asked to discuss these, and to seek over-arching alignment by performing integrated assessments (see also Fig 2 for the design of the table-top simulation exercises). Subsequently, they were asked to jointly review (a selection of) the adjusted advice using the modified WICID assessment framework (see further Waltz et al [29]). Finally, they were asked to draft an additional integrated scientific advice.

### Data collection

Each group and plenary session were led by a facilitator and observed by one (plenary) or two note takers (group sessions). The facilitators ensured that all participants were voiced in the discussions and facilitated the evaluation process of the proposed disciplinary measures. They also facilitated the use of the WICID assessment framework. Note taking was also supported by audio recording of the participants during the group sessions (used only to complete the written note collection). Participants were informed about research procedures and privacy and requested for their consent at the start of each simulation session.

### Data analysis

Outputs from the separate advisory team sessions were reviewed. To gain more insight into the characteristics of the output, itemized lists of recommendations were classified as either guideline (including a reference to the existing guideline) or expert opinion, and an approximate time to result was added to gain more insight into the ability to provide advice in real-time. Discussion topics of integrated deliberations were also listed point by point.

Data were analyzed using an inductive, reflexive thematic analysis (RTA) following Braun and Clarke [30,31]. This approach was selected to enable an in-depth, interpretive analysis of participants’ accounts, with the aim of identifying patterns of meaning across the dataset rather than estimating prevalence or frequency. Analysis proceeded without predefined coding frameworks or quantified inclusion criteria; themes were developed through iterative engagement with the data and reflexive analytic interpretation.

Data collection was participant-led, allowing participants to determine what they considered salient or meaningful. In this context, frequency of mention was not treated as an indicator of importance, as experiences may be implicit, assumed, or difficult to articulate. Quantification of codes or themes was therefore avoided to reduce the risk of misrepresenting the significance of participants’ accounts, consistent with established guidance for reflexive thematic analysis. The disciplinary and domain-overarching HPAI knowledge and know-how gaps identified in the expert interviews in the preparation for and during the simulation exercises were summarized and phrased as potential research questions.

Qualitative analysis of the deliberations of separate and mixed groups, and assessments of the modified WICID frameworks have been evaluated elsewhere (Waltz et al [29]).

### Ethical approval

Our research was part of a bigger research project for which ethical approval was obtained at the DPAS Research Ethics Review Committee, Erasmus University of Rotterdam, the Netherlands (1 February 2024, ETH2324-0189). Each simulation exercise, experts were invited and informed about the purpose and procedures of the simulation exercise in advance via email. Acceptance of the digital invitation was voluntary and considered an informed consent. Additionally, participants were verbally informed about the research procedures and privacy policies at the beginning of each simulation exercise, and were verbally offered the option to opt out. The research data was stored centrally and securely after the simulation exercises.

### Scenarios

The scenario was written by a selection of research authors. Thirty-three experts from various disciplines were interviewed in the preparation for the fictitious but realistic HPAI outbreak scenario. This allowed us to present an outbreak scenario from the perspective of a society as a whole. Validation took place by having the scenario assessed for potential feasibility by two influenza experts.

#### Simulation exercise April 2024.

The following scenario description was prepared: HPAI is found in a small cluster of three pig farms in the northern part of the Netherlands, but not in other animals. Also, two mild human cases are detected, but no secondary human cases. For the following four months, no new cases are detected as in the scenario and swift public health measures are implemented in accordance with Dutch guidelines (e.g., the culling of pigs from contaminated pig farms, a standstill of pigs and related activities within a specified area, source and contract tracing among exposed humans). The fictitious outbreak pushed the Dutch situation from phase 2 to phase 3 in the WHO pandemic influenza classification (see Supplement S1 Fig).

Then, a new variant of H5N1 -nH5N1- is detected in two hospitalized children from the south of the Netherlands with severe respiratory infections, one of whom lives at a pig farm. Source and contact tracing reveals infections with the new variant in six human contacts −3 family members and 3 classmates- and in pigs from 6 out of 20 surrounding pig farms (see also Fig 2). The outbreak elevates the situation to phase 4 of the WHO pandemic influenza classification (Supplement S1 Fig).

#### Simulation exercise May 2024.

**Fig 3:**
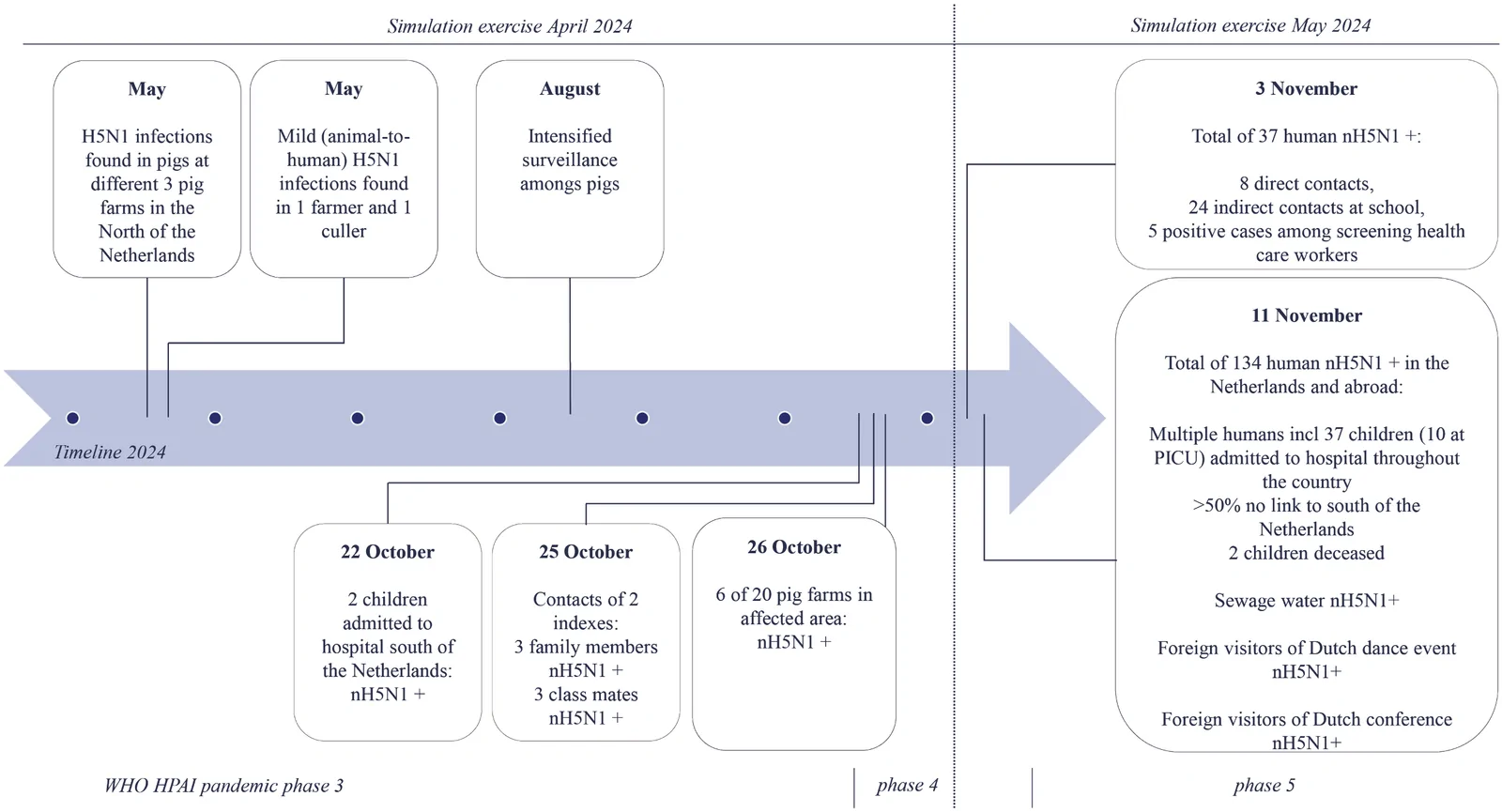
Timeline of a fictitious HPAI scenario of two simulation exercises, April and May 2024, the Netherlands.

The fictitious scenario of the second simulation focused only on human infections and picked up one week later where the first scenario had left off. By this time, a total number of 37 human nH5N1 infections −8 family members, 6 classmates, 18 other students, 5 healthcare workers from a pilot screening- had been confirmed. Thirty-five of the cases had no known exposure to pigs. Despite expanded public health measures are taken in the scenario, the total number of confirmed nH5N1 cases increased to 134 only seven days later, including multiple hospitalizations of children and two deaths. Wastewater surveillance detects the virus in major cities and several international cases linked to events in the Netherlands are reported. The outbreak reaches phase 5 of the WHO pandemic influenza classification (Fig 3, Supplement S1 Table).

## Results

**Fig 4:**
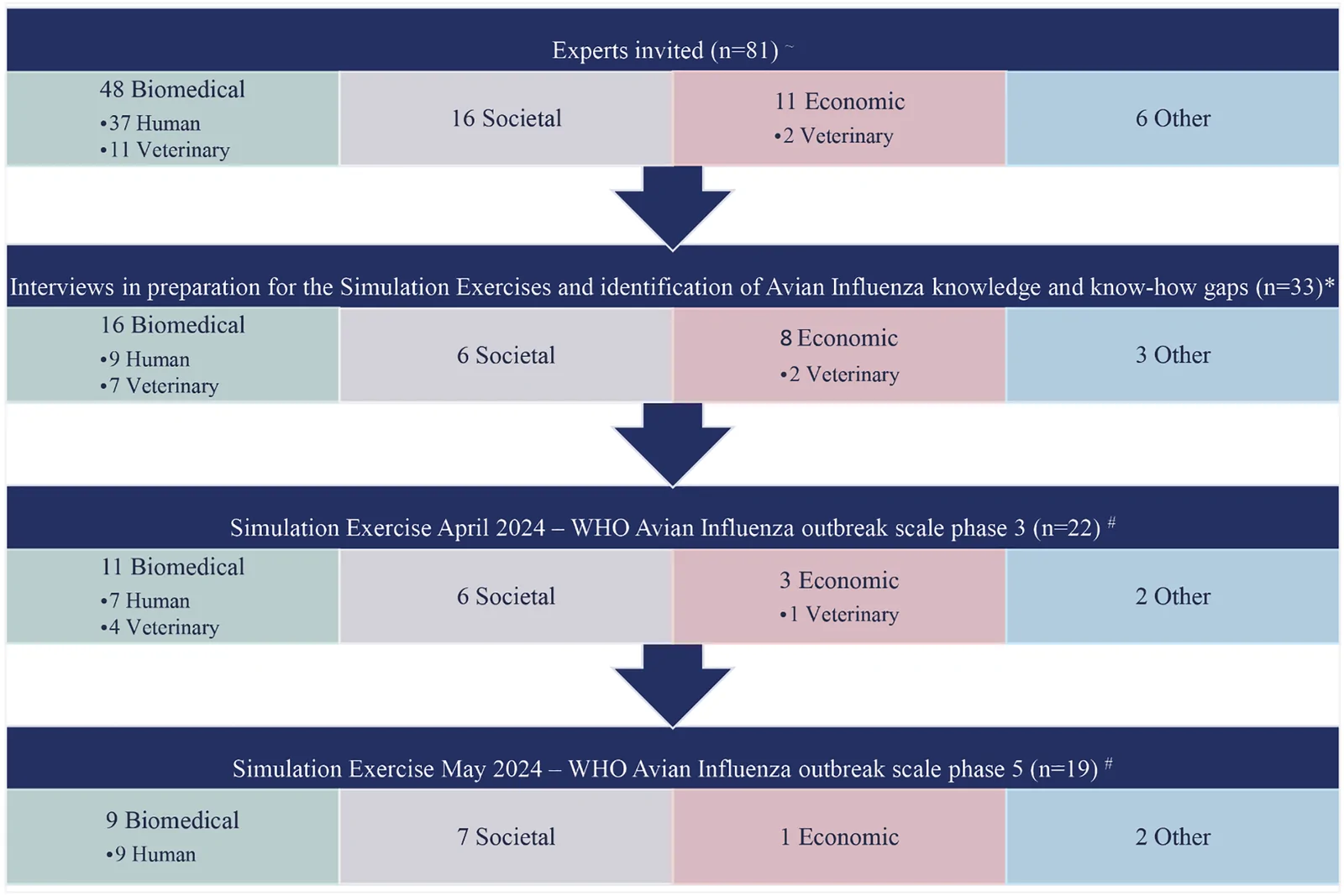
Flow diagram for study participants. * Seventeen of the 33 experts that were interviewed also participated in the simulation exercises. # Eleven (5 biomedical and 7 societal) experts participated in both simulation exercises.

In total, 22 of the 81 invited experts participated in the first simulation exercise (of which 11 participants were assigned to either the biomedical or societal teams, respectively) and 19 experts participated in the second simulation exercise (of which 9 and 10 participants were assigned to the biomedical and societal teams, respectively) (Fig 4). Eleven experts participated in both simulation exercises. The biomedical team consisted of experts, including avian influenza specialists, representing the fields of epidemiology, virology, medical microbiology, infectious disease medicine, public health, infectious disease modelling, family medicine, animal epidemiology, animal modelling of infectious disease transmission, veterinary inspections and poultry health care. The societal team consisted of experts representing the fields of (macro) economics, animal health economics, sociology, health communication, health education, health promotion, public health communication, public health risk communication, risk management, crisis, safety and health, and (government) policy.

### Key knowledge and know-how of separate advice

In the morning sessions, the biomedical and societal teams deliberated separately. The initial recommendations from the separate advice of the April 2024 and May 24 simulation exercises are listed in Tables 1 and 2. The recommendations of the biomedical team were most consistent with recommendations arising from the perspective of outbreak management, including a containment strategy, and limiting the impact of the outbreak itself. The recommendations of societal team were most consistent with recommendations arising from an appropriate communication and information need and limiting the impact of the outbreak and measures.

**Table 1:** The advice provided by the separate biomedical and societal teams during the simulation exercise in April 2024.

		Characteristics of composed advice		
		According to guidelines (advised by)	Expert advice (advised by)	Expected pace of implementation ^~^
Biomedical – human	Source and contact tracing [32]^#^	Biomedical team Societal team		fast
	Active monitoring of high and low risk contacts of the two indexes [32]^#^	Biomedical team Societal team		fast
	Define HPAI as an A1-notifiable disease (diseases in which most forced (public) health measures -including population-based health measures- can be taken by law) [12]^^^		Biomedical team	fast
	Local lock-down of all school children including their families of schools of positive tested children (school closure, work at home, no sports/ entertainment/ restaurants etc.		Biomedical team pro Societal team against	fast
	Active surveillance of all students, teachers and employees of schools of the two indexes		Biomedical team	fast
	Active surveillance of all persons with influenza like illness (ILI) living in the home cities of two indexes		Biomedical team	fast
	Surveillance of health care workers in the hospital of the two indexes		Biomedical team	fast
	Identification and first data collection of critical factors necessary to assess feasibility of a containment strategy of the new pathogen/disease		Biomedical team	fast
Biomedical – veterinary	Culling of all pigs on pig positive tested pig-farms [33]	Biomedical team Societal team		fast
	<3km restricted zone around positive tested pig-farms [33]	Biomedical team Societal team		fast
	<10km transport ban of all pig(-related products) around positive tested pig-farm [33]	Biomedical team Societal team		fast
	<10km transport ban of all animal(-related products) around positive tested pig-farms		Biomedical team	fast
	Composition of swab-team which take swabs of pigs in <3km restricted area		Biomedical team	fast
	Active surveillance in pigs from pig-farms*			
Societal	Prepare potential next steps in the event that measures incorporated in existing medical and veterinary guidelines are no longer sufficient		Societal team^$^	prolonged
	Set up of citizen councils (in schools/cities of index cases). Goals: -to provide honest and factual information -to provide perspectives for action -to prevent social disturbance		Societal team^$^	prolonged
	Set up of information facilities on different levels, dependent of needs identified in citizen councils: -affected areas and national -affected citizens and non-affected citizens		Societal team^$^	prolonged
	Prepare for a more intensive value discussions in society regarding subjects such as animal welfare and intensive livestock farming		Societal team^$^	prolonged
	Set up an active surveillance zone in and around the hometowns of the two indexes to monitor the spread of the virus and social and economic behavior		Societal team^$^	prolonged
	Intensify the consultations among various administrative regarding the virus outbreak		Societal team^$^	fast
Economic	Execute financial support package from funding program (Animal Health Fund) [34]	Societal team		fast
	Prepare potential additional financial funding for affected parties		Societal team^$^	prolonged

# Standard procedures of current public health guidelines regarding highly pathogenic avian influenza [35].

^ Procedure as described in the Dutch Public Health Act (in Dutch ‘Wet Publieke Gezondheidszorg) [36].

$ Measure was only proposed in the event of the worst-case scenario.

* Measure was already introduced as part of the fictitious scenario of the simulation exercise.

~ Measure was considered fast if the expected implementation time, or expected time to first results, of the proposed measure would be within a few days. Otherwise, the implementation time of the measure was considered as prolonged.

**Table 2:** The advice provided by the separate biomedical and societal teams during the simulation exercise in May 2024.

		Characteristics of composed advice		
		According to guidelines	Expert advice	Expected pace of implementation^~^
Biomedical	National lock-down for at least one month (school closure, work at home as much as possible, no indoor sports/ entertainment/ restaurants etc, outdoors more allowed)		Biomedical team	fast
	Non-pharmaceutical measures (NPI’s), including social distancing, use of face masks, and limited number of fixed human contacts. Pending up-scaling of test capacity: persons with symptoms should self-isolate.		Biomedical team	fast
	Up-scaling of test capacity conform guidelines [37].	Biomedical team		fast
	Validate available rapid antigen tests and investigate possibilities of these tests as self-test		Biomedical team	prolonged
	Pharmaceutical measure (PI): consider antiviral drugs as PEP for contacts identified in contact tracing, health care employers, persons with vital professions, persons with symptoms, children		Biomedical team	prolonged
	PI: consider the use of pre-pandemic influenza vaccines for schools with many index cases		Biomedical team	prolonged
	To maintain continuation of care in hospitals: obligatory use of face masks in hospitals, access to PPE essential, easy access to test capacity, and training of intensivists for intensive care of children		Biomedical team	fast
Societal	Prepare impact of best, intermediate and worst-case virus scenarios together with citizens and stakeholders in society		Societal team	prolonged
	Involve the society in an open, honest and clear communication about measures to be taken and the impact of the virus, including: -provide counterfactuals about the effects and costs on society -improve pandemic literacy of the population -provide easy accessible virus knowledge targeted to different groups		Societal team	prolonged
	Collect and monitor data about the spread of the virus, the impact on the population, the general well-being of the population, and the public support of taken measures.		Societal team	prolonged
	Mitigate the negative effects of virus and measures, specifically aimed at mitigating measures for vulnerable and exceptional groups, e.g., informal care givers, parents with vital professions, and young people with unsafe home situations		Societal team	prolonged
	When measures are taken to contain the virus, take into account existing knowledge about support communication and uncertainties.		Societal team	fast
	Start with measures with expected high level of public support -such as working from home, self-isolation in case of ILI symptoms-. In case measures with expected low level of public support -such as school closure- are considered, provide clear considerations and communication as to why these measures are considered necessary.		Societal team	fast
	Within the measures taken, three forms of health must be taken into account: physical, mental and social health. Quantitative data should be collected regarding the impact of the measures -including prevention of deaths-. Impact of the measures and deadlines for this must be weighed against the mutual effects of these three forms of health.		Societal team	prolonged
	Adhere to different outbreak phases for implementation and reconsideration of measures. Evaluate in the current phase after four weeks, including a weekly update of current state and knowledge.		Societal team	prolonged
Economic	Setting up economic support packages for economic sectors.		Societal team	prolonged

~ Measure was considered fast if the expected implementation time, or expected time to first results, of the proposed measure would be within a few days. Otherwise, the implementation time of the measure was considered as prolonged.

### Topics in separate advice leading to conflicting recommendations

Without the teams explicitly naming their focus, we observed in both simulation exercises that the two separate teams initially worked particularly with one of the two policy goals of the Health Ministry’s request for outbreak management advice. The biomedical team focused particularly on the policy goal ‘guard healthcare access for the entire population’, including sub questions such as what (public health) options are available to reduce risks. The societal team focused mainly on the other policy goal ‘social and economic continuity and vitality,’ including related sub questions. As a result, the separate pieces of advice appeared to be complementary at first glance. However, school closure -as proposed by the biomedical team- was one of the topics that was discussed and explicitly excluded from the list of possible interventions by the societal team. Because one expert team’s itemized list was not discussed by the other expert team, it was not clear whether other topics or proposed measures resulted in conflicting recommendations during the separate advice sessions. In particular since the societal team also recommended measures that could be considered as part of the biomedical expertise (such as set up a surveillance zone to monitor the spread of the virus).

### Characteristics of separate advice leading to discord in the process of advising

**Fig 5:**
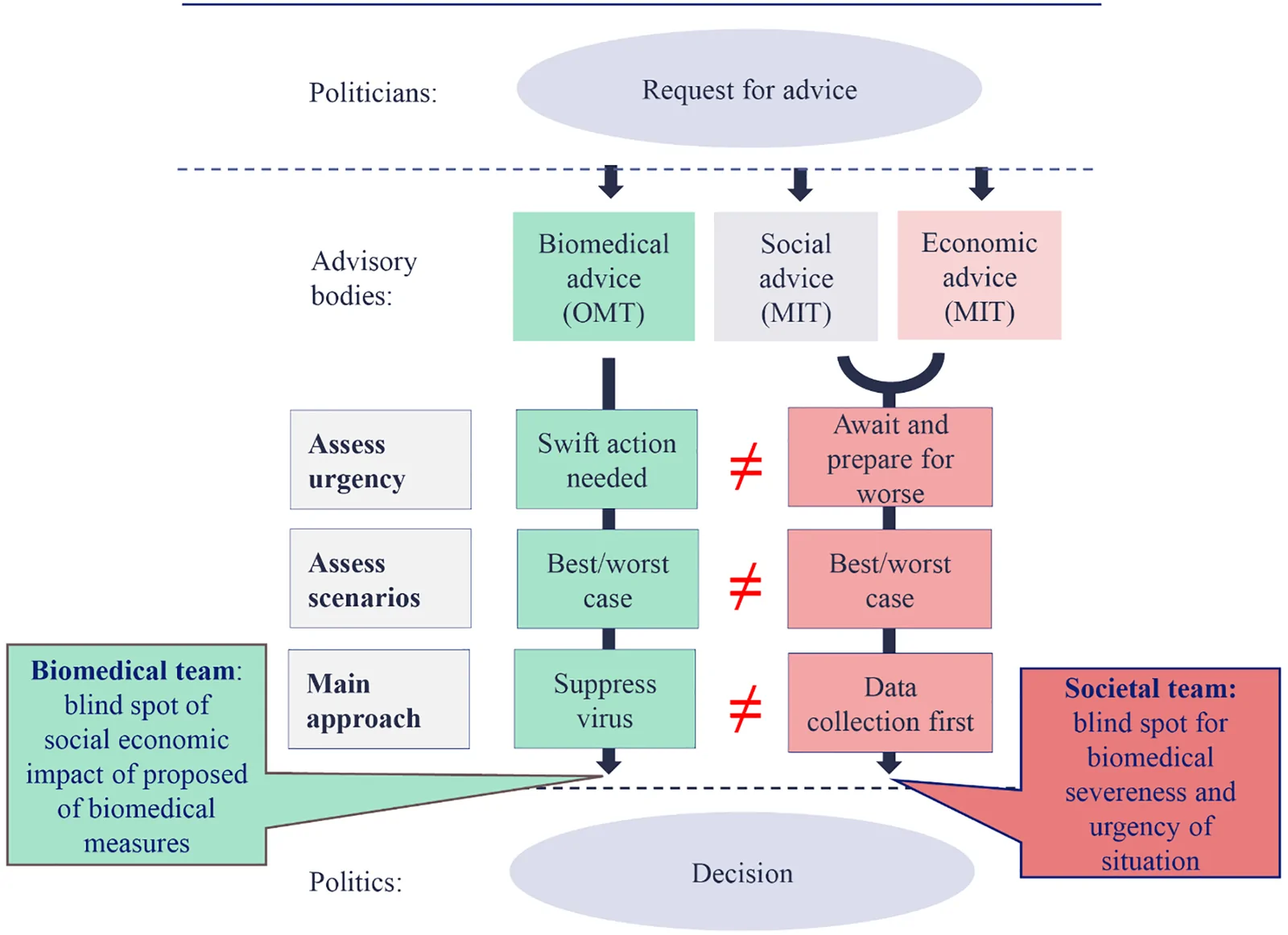
Discord factors observed in separate biomedical and societal advice during the morning sessions of the fictitious simulation exercises in April and May 2024.

When reviewing the advice from each expert group in a plenary forum, several differences were noted. First, the starting point for the medical, veterinary and economic experts was captured in existing guidelines or funding programs, but the societal experts could not rely on existing guidelines. As a result, the pace of execution of the intended measures differed considerably between the fields. Second, without a biomedical interpretation, the (potential) severity of the situation was not clear to the societal experts. This forced them to compose a best and worst-case scenario themselves, which led to different best- and worst-case scenarios between the expert groups during the first simulation exercise. The assessment of severity and the required pace of action also differed between the two groups. Third, blind spots were observed. By prioritizing the goals most important to their own expert team -such as virus suppression for the biomedical team and limiting social side effects for the societal team-, public health measures proposed by one expert team seemed to overlook the potential impact or consequences in the other expert team’s field. Finally, in the deliberations, experts referred to negative experiences from the COVID-19 pandemic (including public health measures taken during the pandemic) and included these in their decision making. We have summarized the observed discord factors in Fig 5.

### Key themes contributing towards alignment in advice and process

After the plenary review of the morning sessions, the experts were mixed in two or three groups respectively. The main discussion points, of the simulation exercise in April and May 2024 are listed in Table 3.

**Table 3:** The topics of the integrated deliberations during the afternoon sessions of the fictitious simulation exercises in April and 2024.

	Discussion points during integrated deliberations
April 2024 (three groups)	-different sense of urgency and pace of action between the two separate advices -focus of biomedical team is containment, focus of societal team is social impact and support -focus of biomedical team is containment, societal team needs explanation why prompt action is necessary -proportionality of extensiveness of proposed biomedical measures -blind spot of biomedical team: the lack of considering social and economic impact of proposed biomedical advice (such as school closure) -blind spot of societal team: the ability to acknowledge the potential severe consequences of the newly discovered virus and need for prompt action -use of concealing terminology for measures including its impact and potential negative connotation (“standstill” instead of lockdown
May 2024 (two groups)	-Different sense of urgency -proportionality and expected social impact of proposed biomedical measures

We observed that the identified blind spots in these participating groups, the inconsistencies in the approach and characteristics of the proposed public health measures in the separate advising were explored during the integrated deliberations. In all mixed groups, the deliberations included the difference in the assessment of the situation. Recognizing that this was part of the biomedical group’s area of expertise, members of the societal team sought clarification from the biomedical team as to why they were recommending rapid action despite a limited number of cases with relatively mild disease. Members of the biomedical team were keen to provide additional explanations of the potential consequences of a worst-case scenario, and why it was important to act swiftly to preserve the window of opportunity to take effective countermeasures. The integrated deliberations facilitated the identification of underlying disciplinary assumptions and that transparency helped clarify why certain advice was being given.

School closure was one example that was discussed at length. Members of the biomedical team considered the outbreak of a new variant involving children with very little herd immunity to be a very severe situation and preferred to act swiftly and rigorously to prevent the outbreak to evolve in a larger outbreak, while members of the societal team seemed not impressed by the severity due to the limited cases identified and considered school closure to be too impactful, given lessons learned from the COVID-19 pandemic.

*“Another point that struck us in particular was the closure of schools as a measure. This is a very extreme measure in the socio-economic world. It is a costly measure in social and economic terms if it is to continue for a long time. The advice now gives the impression that this has been taken lightly.”*

Discussing this difference in sense of urgency and the proportionality of school closure allowed the identification of blind spots on both sides. For example, an explanation of the potential severity of the outbreak itself clarified why the biomedical team recommended such stringent and urgent measures.

*Member of the societal team in a mixed deliberation: “If we had known earlier how serious it was from the biomedical team’s point of view”*

On the other hand, the socioeconomic perspective prompted the biomedical team to reconsider proposed measures after explaining their potential socioeconomic impact.

*Member of the societal team in a mixed deliberation: “You are recommending measures that could cause significant mental and social damage. It is important to carefully consider whether to include them in the package.”*

The mixed deliberation led to a better understanding of the underlying rationale from the different fields of expertise. The participants were willing to reconsider the proposed measures or to jointly discuss additional measures to minimize the consequences of the outbreak or the measures.

**Fig 6:**
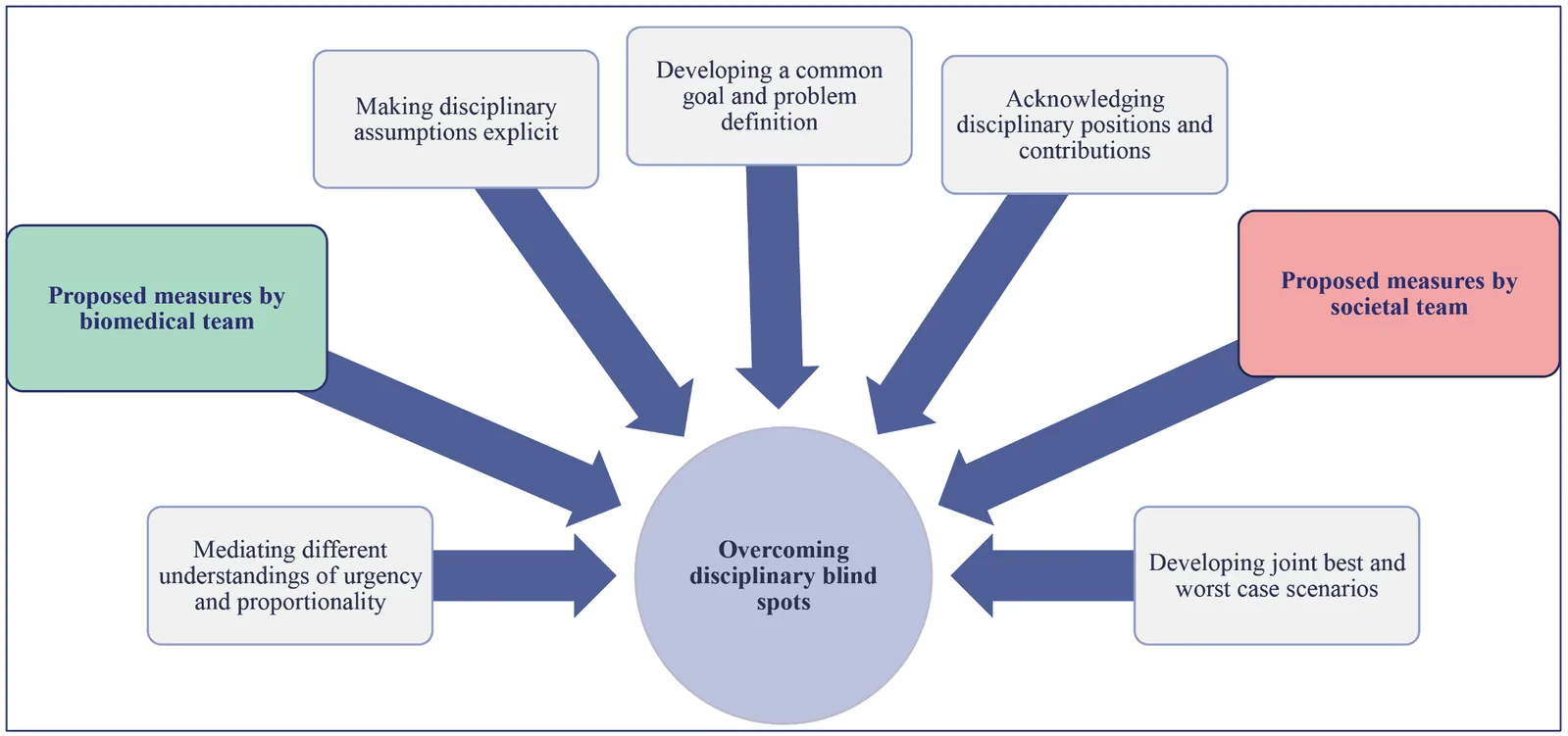
Key themes during the integrated deliberations as potential enablers to overcome potential for conflicting expert advice.

Figure 6 summarizes key factors enabling the integrated decision making. However, the speed and success of these deliberations differed between the mixed groups of experts. Particularly in the April session, one group developed a mutual understanding quickly and smoothly, while another group struggled to find common ground.

Remarkable was that the variety of discussion points was less extensive during the second simulation exercise compared to the first exercise.

### HPAI knowledge and know-how gaps

As progressive knowledge and know-how may contribute to HPAI preparedness, we sought to identify biomedical, societal, economic and domain-overarching gaps in the preparation for and during our simulation exercises. We observed discordance between the level of knowledge and know-how gaps: most of the identified biomedical gaps were related to fundamental HPAI research and to available HPAI knowledge that has not (yet) been incorporated into guidelines, while societal gaps were mainly related to the lack of availability of general response guidelines, surveillance structures and infrastructures in case of any new outbreak (see supplement S1 Table). Domain-overarching gaps were also more related to general domain-overarching outbreak response gaps than to those specific to HPAI outbreak response. Those that surfaced during the integrated session were primarily related to a lack of cooperation or alignment in the outbreak response. We have listed the domain-overarching gaps identified in the preparation and during the simulation exercise in Table 4.

**Table 4:** Domain-overarching knowledge and know-how gaps identified by participants in the interviews and observed by participants or research team during the HPAI simulation exercises in April and May 2024, the Netherlands.

		Identified knowledge and know-how gaps
Domain-overarching	Knowledge/ know-how	Can we align response guidelines, structures and organization across disciplines so that an outbreak response acts as a whole, is as complete as possible and proceeds at a similar pace?
	Knowledge/ know-how	Can we optimize international (One Health) coordination structures to act as promptly as possible in the case of cross-border threats?
	Knowledge/ know-how	How can policymakers and scientists be made aware of how best to cooperate?
	Knowledge/ know-how	Can we design a structure to involve other experts such as tech, science, IT or communication experts?
	Knowledge/ know-how	How can Dutch people be instructed to prepare for a lock-down and to have basic supplies (canned food/drinking water) at home for a certain period of time?
	Knowledge/ know-how	How can we best use data from lessons learned from previous success stories?
	Knowledge/ know-how	Can we prepare for scenarios where certain supplies are scarce, and how can we best use those supplies?
	Know-how	Can we identify roles, responsibilities and point of contacts from all organizations (including volunteers) to avoid duplication of work and loss of valuable time and personnel?
	Know-how	How can we create social support for impactful measures to prevent a major outbreak when the case load is still low?
	Know-how	Can we design a communication structure between OMT and MIT in the event of a (pandemic) outbreak?
	Know-how	Can we design a structure between OMT and MIT how to draft mutual best, worst or most likely case scenarios?

## Discussion

The simulation exercises demonstrated that different deliberation topics and a different interpretation of the acute outbreak situation can lead to considerable discrepancies between the separate scientific advisory teams. Discrepancies were found both between the separate pieces of expert advice as well as in the process of advising. These discrepancies included blind spots towards other expert fields, a different sense of urgency and a different sense of proportionality among the participating experts. During integrated deliberations, the different interpretations converged, which seemed to be a prerequisite for creating space to resolve the observed conflicting topics and characteristics of the advice process.

Multidisciplinary team meetings, providing space for multiple perspectives and consensus building, are well established in many complex clinical situations and are part of standard hospital care settings. Although these teams are often considered as multidisciplinary within the biomedical field, it is not uncommon for disciplines such as social work to be included in such teams. Social perspectives on issues are therefore often secured in such teams. Extending this concept to complex public health situations seems only comprehensible. In an advisory report (2022) of the Royal Netherlands Academy of Arts and Sciences and the Partnership Contribution High-Level Implementation Plan III 2024–2030 of the WHO, collaboration across disciplines to be adequately prepared for large-scale infectious outbreaks is recommended [38,39]. The European Commission already secured experts from various disciplines in their advisory committee for the occurrence and recognition of a public health emergency at Union level in their regulations about serious cross-border threats (2022/2371) [40]. However, there are no clear guidelines how to collaborate across disciplines in pandemic settings when the certainty of scientific evidence can be low and decision-makers need actionable advice to act swiftly. Existing evidence-to-decision/recommendation frameworks such as the Grading of Recommendations Assessment, Development and Evaluation, known as GRADE, assists the process of grading quality (or certainty) of evidence and strength of recommendations [41]. GRADE is considered a standard approach for guideline development in biomedical areas, but not specifically in public health. A GRADE Evidence to Decision framework for health system and public health decisions was developed in 2018, but does not specifically describe the multidisciplinary collaboration, nor does it necessarily produce an integrated advice [41]. The WHO INTEGRATE evidence-to-decision framework (2019) was developed as more holistic approach, but seemed limited adopted by national advisory structures thus far [42].

In our simulation exercises, the diversity of discussion points in the integrated deliberations was less extensive in the second simulation exercise. Possibly, the groups dynamics were different in the second simulation session due to the composition of different teams, but perhaps the interpretation of the severity of the outbreak was more comparable among all experts due to the strongly increased number of (hospitalized) cases -in particular youngsters- in the scenario. A best and worst-case scenario was also presented before the separate deliberations in the second simulation session, creating a similar scenario base for both groups. In addition, several experts had participated in the first simulation exercise, which could have influenced the level of basic knowledge of the participants in the second simulation exercise. We believe that the settings of the second simulation exercises contributed to a more aligned interpretation and goal setting among the participating experts. However, it is arguable whether these deliberations should take place during an acute outbreak situation. Improvement of basic knowledge and identification of blind sports should preferably be overcome in the preparation phase of potential new outbreaks, rather than during a crisis, as this runs the risk of delaying the ability to provide advice in a timely manner. The continuation of simulation exercises with participants from different disciplines -as suggested by the WHO in the preparation of large-scale influenza outbreaks- may be a good way forward to further train the collaborative development of advice [43].

The legacies of the COVID-19 pandemic appeared to be both a great source of acquired pandemic-related know-how and knowledge as well as a barrier to interpret the simulation outbreak without the negative COVID-19 sentiments. As for children, the negative effects of the public health measures taken were considered to be more impactful than the disease during the COVID-19 pandemic by the societal expert group. Therefore, the proposed public health measures affecting children in the outbreak simulation led to an extensive proportionality discussion during the integrated sessions. A discussion of the precautionary approach, or the underlying principles and values of the considerations of advice, could have assisted in this process of alignment. This was not included in the design of the simulation exercise and was therefore not structurally addressed. School closure should be weighed thoroughly as there are still many uncertainties about its health, economic and social impact [44]. However, the school closure data collected in COVID-19 may not be applicable to an influenza outbreak once children are at risk for severe disease, as in our fictitious simulation exercise, or during the 1918 influenza pandemic (Spanish flu) or the 2009 H1N1 influenza outbreak [44,45]. During the latter outbreak, several schools in the United States were closed for a variety of reasons, including high student absenteeism [46]. Pre-simulation framing or context stripping might be considered for future research to minimize potential bias caused by experiences in previous outbreaks.

Preferably, knowledge and know-how gaps are minimized to optimize pandemic preparedness. To contribute to the optimization process, we have tried to identify current knowledge and know-how gaps specific to avian influenza in the preparation for and during our fictitious simulation exercises. Although many goals have already been achieved in the Netherlands, a few goals of the WHO checklist for respiratory pathogen pandemic preparedness planning were identified as gaps [43]. Optimization and incorporation of how to achieve equity of (potentially) scarce resources -such as PPE or vaccines- should be prioritized now that the avian influenza threat is considered as low. In particular as the current 2024 Mpox outbreak demonstrate that equitable access to vaccines is still challenging [47].

To align with the biomedical level of outbreak preparedness, the societal sciences should prepare for swift action when needed, for example by developing guidelines or -as suggested in previous research- ‘field-ready methodological tools’ [48]. The assessment framework developed by the MIT (July 2024) -a tool that can be used to identify the effectiveness and societal impact of measures, together with the key dilemmas and underlying values- is a major step in this process [49].

In addition to the observed misalignment in urgency and potential scenarios, the different implicit policy goals set by the separate teams seemed to have contributed to the divergence of proposed measures. Additionally, the different underlying perspectives of the separate teams on the proposed measures—outbreak management and limiting the outbreak’s impact versus communication and information strategies and limiting the impact of measures—seemed to enhance the misalignment. Therefore, it seems valuable to systematically structure separate deliberations in future research to minimize potential misalignment and blind spots caused by different policy goals, perspectives, or expectations of potential scenarios. To explore how to minimize disciplinary blind spots, future research should also focus on studying different methods of interaction between the two teams.

Our simulation exercises had some limitations. First, due to the design of the simulation exercise, the participants had only limited time to deliberate, and craft separate or integrated advice. Also, conflicting and aligned perspectives were not systematically assessed during the simulation exercise, and an aligned structure of the deliberations of the separate teams was not included. This probably influenced the accuracy of the advice formulated. Future research should incorporate a systematic assessment of potential conflicting topics and ensure alignment in the deliberation processes of the separate teams.

Second, though the scenario was well prepared and certain information will be unknown during an actual outbreak and needs to be investigated -such as the extent of the outbreak, who is most at risk, etc-, some information was unknown in our scenarios that would be known in real life -such as the number of doses of anti-viral medication. Veterinary cases were included in the second simulation exercise to a limited extent for simplicity purposes, but would not be neglected in a real outbreak. Also, the scenarios included biomedical, social and economic perspectives, but it included only limited modeling perspectives and no perspectives from other disciplines. It did not include simulated real-time data for example. Recommendations such as school closure, were drafted in the absence of this relevant data. In addition, the experience of external attention and pressure -e.g., from politicians, foreign countries, the media- is challenging to incorporate into a simulation exercise but cannot be neglected in real-life decision making. These limitations may have influenced the deliberations and advice formulated. We recommend incorporating a fictitious dashboard into future simulation exercises to enable modeling and recommendations based on real-time (fictitious) data.

Third, the results of these exercises were both exercise and participant dependent. A different scenario -or another pandemic phase of this scenario- and/or different participating experts could have led to different group dynamics, different communication styles and/or different perceived authority or hierarchy within the teams and may have led to different results. To include normative guidance, it is recommended to include ethicists and legal experts in future research.

Finally, the knowledge gaps described are only identified for the context of fictitious avian influenza exercise in the Netherlands and are not exhaustive. For effective pandemic preparedness, it would be useful to provide an overview of the urgency of the gaps identified in follow-up research.

Despite the limitations and the fictitious scenario, the identified misalignments in the advisory process, structures and knowledge and know-how gaps reflect current challenges in the scientific advisory process. The conflicting perspective of fictitious recommendations should also be taken seriously, as it provides food for thought on how to prepare for an actual outbreak of concern. The Dutch national advisory structures were used as the basis for our research project. However, since multidisciplinary perspectives should be incorporated into any pandemic advisory structure, we think our results regarding the integration of scientific advice can be generalized and are useful for other countries seeking to optimize outbreak management.

## Conclusion

Our simulation exercises of a fictitious acute HPAI outbreak in the Netherlands suggest that an integrated approach to pandemic advice combining biomedical and socio-economic expertise is challenging, but may have value in overcoming conflicting advice and discrepancies in the process of advice. Even though our research was national oriented, and participants and group dynamics could have influenced our results. Further exploration using additional scenario exercises is therefore needed, to determine whether and under what circumstances combined advisory bodies could add value. These outcomes will be beneficial for the Netherlands and beyond. Most of the identified knowledge and know-how gaps should be addressed in the current pre-pandemic phase, to reduce the loss of valuable time during an outbreak of (potential) pandemic threat. Pre-pandemic training and simulation exercises may help train such collaboration to prepare for potential pandemics.

## Supporting information

S1 Fig Supplement Fig 1: WHO description of pandemic phases in the event of an animal influenza pandemic [50]. (DOCX)

S1 Table Supplement Table 1: Disciplinary knowledge and know-how gaps identified in preparation for and during the HPAI simulation exercises in April and May 2024, the Netherlands. (DOCX)
