# Supplementary material for: Exploring integrated advice and avian influenza knowledge gaps by using a Highly Pathogenic Avian Influenza outbreak simulation
Source: PLoS One. 2026 Aug 26;21(8):e0354920. doi: 10.1371/journal.pone.0354920 (PMC13514051; doi:10.1371/journal.pone.0354920)
Supplement: Supplementary file 1 [file pone.0354920.s001.docx]

**S Figure 1**

| **WHO pandemic phase description** | |
| --- | --- |
| Phase 1 | No animal influenza virus circulating among animals has been reported to cause infections in humans. |
| Phase 2 | An animal influenza virus circulating in domesticated or wild animals is known to have caused infection in humans and is therefore considered a specific potential pandemic threat. |
| Phase 3 | An animal or human-animal influenza reassortant virus has caused sporadic cases or small clusters of disease in people, but has not resulted in human-to-human transmission sufficient to sustain community-level outbreaks. |
| Phase 4 | Human-to-human transmission (H2H) of an animal or human-animal influenza reassortant virus able to sustain community-level outbreaks has been verified. |
| Phase 5 | The same identified virus has caused sustained community level outbreaks in two or more countries in one WHO region. |
| Phase 6 | In addition to the criteria defined in Phase 5, the same virus has caused sustained community level outbreaks in at least one other country in another WHO region. |
| Post-peak period | Levels of pandemic influenza in most countries with adequate surveillance have dropped below peak levels. |
| Possible new wave | Level of pandemic influenza activity in most countries with adequate surveillance rising again. |
| Post-pandemic period | Levels of influenza activity have returned to the levels seen for seasonal influenza in most countries with adequate surveillance. |

Supplement Figure 1*: WHO description of pandemic phases in the event of an animal influenza pandemic (39).*
