# Supplementary material for: Exploring integrated advice and avian influenza knowledge gaps by using a Highly Pathogenic Avian Influenza outbreak simulation
Source: PLoS One. 2026 Aug 26;21(8):e0354920. doi: 10.1371/journal.pone.0354920 (PMC13514051; doi:10.1371/journal.pone.0354920)
Supplement: Supplementary file 2 [file pone.0354920.s002.docx]

**S Table 1**

Supplement Table 1: *Disciplinary knowledge and know-how gaps identified in preparation for and during the HPAI simulation exercises in April and May 2024, the Netherlands.*

|  |  | **Identified knowledge and know-how gaps** |
| --- | --- | --- |
| Biomedical | knowledge | What are biological properties/virulence factors of avian influenza which provide insight into the mutations that are the most threatening? |
|  | knowledge | Can we further develop and optimize animal models to study vaccine efficacy to facilitate the extrapolation of the data from pre-clinical studies to clinical studies? |
|  | knowledge | Can we generate novel vaccines that are able to overcome the challenges of antigenic diversity to prevent low immunogenicity of available vaccines in case of a new variant? |
|  | knowledge | Can we extend our limited sight on the (development of) influenza- and avian influenza variants among mammals, including pigs while early identification is first line defense in case of a new disease outbreak? |
|  | knowledge | Can we extend our limited knowledge about potential advantages of air treatment systems on airborne pathogens? |
|  | knowledge | Can the expected concrete impact of an intervention be modelled? In particular, can the closure of different parts of the society (e.g. schools) to contain the pathogen be modelled with acceptable accuracy for the given pathogen/outbreak at a given time, including the very early onset of the outbreak? |
|  | Knowledge/  know-how | Can livestock sectors -in addition to the poultry sector- increase their preparedness by developing (economic) support packages in case of an AI outbreak? |
|  | Know-how | Can available municipal crisis structures that deploy psychosocial help in the event of a crisis also be effectuated in case the crisis still primarily concerns animals? |
|  | Know-how | Can current guidelines anticipate in:  -the implementation of the registered pre-pandemic avian influenza vaccine?  -the use of antiviral medication as P(r)EP and large scale swift prescription/start treatment (<48 hours)?  -potential emergence of viral resistance, and treatment strategies with lowest risk of mutant selection?  -the most effective distribution of means in case of scarcity? Such as limited access to PPE, antiviral medication and/or (pre-pandemic).  -Upscaling scenarios in case availability of (culling)materials and -personnel are lacking?  - Upscaling scenarios in case availability of test/diagnostic (PCR) materials is still lacking?  -Continuation/reopening scenarios of society in case pharmaceutical interventions (eg vaccins) are not available (yet)?  -a reconsideration of the proportionality of isolation and quarantine duration in case of a major (long lasting) outbreak? |
| Social | Knowledge | How can we extend the limited data about support of and adherence to public health measures among farmers? |
|  | Knowledge | What assessment and decision guidelines or frameworks should be in place to weigh different preventive measures and related issues from an ethical perspective? |
|  | Knowledge | What is the effect of behavioral measures on pathogen transmission and spread? |
|  | Knowledge | What is the effectiveness of different risk communication? |
|  | Knowledge | What is the social and psychological impact of different measures? |
|  | Knowledge/ know-how | What is required to train communications staff (at ministries) to communicate outbreak information and public health measures to a wide audience, also to counter misinformation? |
|  | Know-how | How to set up a solid structure to monitor both the impact of public health measures and detect their consequences in a timely manner? |
|  | Know-how | How can key ethical principles for different phases of an outbreak be operationalized? |
|  | Know-how | How can we ensure that all elements of the existing crisis guidelines are being applied? |
|  | Know-how | How can citizens be involved in short term decision making? |
|  | Know-how | Can we design an infrastructure how to reach and monitor underserved citizens? |
|  | Know-how | Can simulation exercises of different scenarios contribute to a clarification of the aims of social policy? |
| Economic | Knowledge | Can we extend the limited available data about the drivers why citizens interrupt consumption patterns during an outbreak/lockdown? |
|  | Knowledge | Can we extend the limited data regarding the complex decision making scenarios who, when and to which extend is eligible for (financial) support packages? |
|  | Knowledge/ know-how | Why is there no fund for Dutch people who have been diagnosed with a notifiable disease and who have a package of public health measures imposed on them, where they can claim a support package, as is the case in the veterinary sector-? |
|  | Know-how | Can we design an infrastructure how to reach only those affected? And is it fair to compensate people who are affected but not those who took the measures to be resilient? |
